# Association between exposome score for schizophrenia and functioning in first-episode psychosis: results from the Athens first-episode psychosis research study

**DOI:** 10.1017/S0033291721004542

**Published:** 2023-04

**Authors:** Gamze Erzin, Lotta-Katrin Pries, Stefanos Dimitrakopoulos, Irene Ralli, Lida-Alkisti Xenaki, Rigas – Filippos Soldatos, Ilias Vlachos, Mirjana Selakovic, Stefania Foteli, Ioannis Kosteletos, Nikos Nianiakas, Leonidas Mantonakis, Emmanouil Rizos, Konstantinos Kollias, Jim Van Os, Sinan Guloksuz, Nikos Stefanis

**Affiliations:** 1 Department of Psychiatry, University of Health Sciences Ankara Diskapi Training and Research Hospital, Ankara, Turkey; 2 Department of Psychiatry and Neuropsychology, School for Mental Health and Neuroscience, Maastricht University Medical Center, Maastricht, the Netherlands; 3 First Department of Psychiatry, National and Kapodistrian University of Athens Medical School, Eginition Hospital, Athens, Greece; 4 Psychiatric Clinic, 414 Military Hospital of Athens, Penteli, Greece; 5 Second Department of Psychiatry, National and Kapodistrian University of Athens Medical School, ‘ATTIKON’ University Hospital, Athens, Greece; 6 Department of Psychiatry, UMC Utrecht Brain Centre, University Medical Centre Utrecht, Utrecht University, Utrecht, The Netherlands; 7 Department of Psychosis Studies, Institute of Psychiatry, Psychology & Neuroscience, King's College London, London, UK; 8 Department of Psychiatry, Yale University School of Medicine, New Haven, CT, USA

**Keywords:** Cannabis use, childhood trauma, environment, functioning, outcome, psychosis

## Abstract

**Background:**

Evidence suggests that environmental factors not only increase psychosis liability but also influence the prognosis and outcomes of psychotic disorders. We investigated temporal and cross-sectional associations of a weighted score of cumulative environmental liability for schizophrenia – the exposome score for schizophrenia (ES-SCZ) – with functioning in first-episode psychosis (FEP).

**Methods:**

Data were derived from the baseline and 1-month assessments of the Athens FEP Research Study that enrolled 225 individuals with FEP. The Global Assessment of Functioning (GAF) and the Personal and Social Performance Scale (PSP) were used to measure social, occupational, and psychological functioning. The ES-SCZ was calculated based on the previously validated method.

**Results:**

ES-SCZ was associated with the total scores of GAF and PSP at baseline and 1-month assessments. These findings remained significant when accounting for several associated alternative explanatory variables, including other environmental factors (obstetric complications, migration, ethnic minority), clinical characteristics (duration of untreated psychosis, symptom severity, previous antipsychotic use), and family history of psychosis, demonstrating that the association between ES-SCZ and functioning is over and above other risk factors and cannot be explained by symptom severity alone. Functioning improved from baseline to 1-month assessment, but no significant ES-SCZ-by-time interaction was found on functioning, indicating that functioning changes were not contingent on ES-SCZ.

**Conclusions:**

Our findings suggest that rather than a predictor of functional improvement, ES-SCZ represents a stable severity indicator that captures poor functioning in early psychosis. Environmental risk loading for schizophrenia (ES-SCZ) can be beneficial for clinical characterization and incorporated into transdiagnostic staging models.

## Introduction

Psychosis spectrum disorder is a complex syndrome with a wide variety of clinical representations that follow diverse illness trajectories and outcomes after the first episode of psychosis (FEP), ranging from complete remission to chronic course and significant functional impairment (Guloksuz & van Os, 2018). Functional impairments including social, occupational, and independent living activities emerge during the early phases of psychosis and functional remission and recovery rates vary over time (Chang et al., 2016, 2018; Kuzman et al., 2020; Rosengard et al., 2019; Strålin, Skott, & Cullberg, 2019; Verma, Subramaniam, Abdin, Poon, & Chong, 2012). Therefore, major goals of FEP management include not only relapse prevention at the symptom level but also functional remission and quality of life improvement.

Prognosticating psychosocial functioning trajectories can help improve outcomes by early individualized interventions targeting functional improvement. To this end, efforts have recently been made to develop multivariate models prognosticating functional outcomes, as well as short-term clinical remission (Koutsouleris et al., 2018; Soldatos et al., 2021). Clinical features such as duration of untreated psychosis (DUP), negative symptomatology, and neurocognitive status are associated with functioning at onset and functional recovery over the course of FEP (Kuzman et al., 2020; Mezquida et al., 2017; Penttilä, Jääskeläinen, Hirvonen, Isohanni, & Miettunen, 2014; Peralta et al., 2021; Santesteban-Echarri et al., 2017; Seddon et al., 2016). Evidence suggests that environmental exposures, which form a complex and dynamic mutually interconnected network (Guloksuz, van Os, & Rutten, 2018), are directly as well as indirectly associated with outcomes in FEP. For instance, childhood trauma is significantly associated with reduced global functioning and more severe clinical symptoms among individuals with FEP (Aas et al., 2016). Cannabis use in individuals with FEP has been linked to specific domains of social role functioning, such as economic and social activities, after 2 years of follow up (Faber et al., 2012). Evidence also suggests that modifying these factors, for example reducing cannabis use, could prevent worse outcomes in FEP (Schimmelmann et al., 2012).

A growing body of evidence suggests that a cumulative environmental liability score of schizophrenia can be beneficial for predicting transition to psychosis in at-risk for psychosis populations (Padmanabhan, Shah, Tandon, & Keshavan, 2017), as well as capturing stress sensitivity to adverse life events (Pries et al., 2020a), identifying schizophrenia (Pries et al., 2021b), and psychosis risk stratification in the general population (Guloksuz et al., 2020). The findings of a recent cross-sectional analysis replicated in two independent samples showed that a weighted score of cumulative environmental liability for schizophrenia, the exposome score for schizophrenia (ES-SCZ), was associated with global functioning in patients diagnosed with schizophrenia spectrum disorder (Erzin et al., 2021). However, these findings were based on cross-sectional analyses in individuals diagnosed with schizophrenia. To our knowledge, no longitudinal study analyzed the temporal association between ES-SCZ and clinical outcomes including symptom severity and clinical functioning in FEP.

In this study, our aim was to investigate, for the first time, temporal and cross-sectional associations of ES-SCZ with symptom severity and functioning that covers social, occupational, and psychological domains. The use of a prospective cohort of individuals with FEP that collected detailed information on clinical features, functional measures, and environmental exposures over a year provided a unique opportunity to minimize the confounding of prior treatment when analyzing ES-SCZ (environmental predisposition proxy for psychosis) as an indicator of poor functioning.

## Methods

### Study population and design

The Athens FEP Research Study enrolled patients with FEP consecutively from the emergency departments of five different psychiatric hospitals (Eginition University Hospital, 414 Military Hospital, Atticon University Hospital, Sismanoglion General Hospital and Sotiria General Hospital). Inclusion criteria were the presence of FEP at age 16–45 years and a maximum of 2 weeks of exposure to antipsychotics. Individuals with psychotic disorders due to another medical condition or acute intoxication, IQ < 70, developmental disorders, and kinship with an enrolled participant were excluded from the study. All participants were screened using the diagnostic interview for psychosis (Castle et al., 2006). The sample was collected between March 2015 and March 2020. Of 279 identified individuals eligible for the study, 225 were included. Interviews at baseline, 1-month, and 1-year were conducted by clinically qualified researchers with psychiatric or neuropsychological specializations who were also formally trained by authorized trainers to apply the assessment instruments. In expert consensus meetings, involving the principal investigators and the research associate assigned with each case, ICD-10 (World Health Organization, 1992), DSM-IV-TR (American Psychiatric Association, 2000) and DSM-5 (American Psychiatric Association, 2013) diagnoses were determined at 1-month and 1-year follow up visits. The clinical, environmental, and other psychometric measurement tools were compatible with those used in the European Network of National Schizophrenia Networks studying Gene-Environment interactions (EU-GEI, 2010). The Research Ethics Committees of the five participating hospitals approved the study protocol. Signed informed consent was collected from all individuals. More details of the study design were provided elsewhere (Xenaki et al., 2020).

### Functioning outcomes

Consistent with previous work (Erzin et al., 2021), the primary outcome of this study was global functioning that was assessed using the Global Assessment of Functioning Scale (GAF) and the Personal and Social Performance Scale (PSP). The GAF scale is a well-known standard rating instrument to measure social, occupational, and psychological functioning (Endicott, Spitzer, Fleiss, & Cohen, 1976). GAF scores range from 1 to 100. Higher scores in the GAF total score reflect an increase in mental health and coping ability, and lower scores reflect a decrease in mental health and coping capacity (Pedersen & Karterud, 2012; Pedersen, Hagtvet, & Karterud, 2007). The PSP measures functioning across four domains: social useful activities, personal and social relationships, self-care, and disturbing and aggressive behavior. The PSP total score is a single overall rating from 1 to 100. Higher scores in the PSP total score indicate better personal and social functionality (Morosini, Magliano, Brambilla, Ugolini, & Pioli, 2000).

### Exposome score for schizophrenia

With exception of hearing impairment, which was not collected in the Athens FEP Research Study, the assessments and definitions of environmental exposures were consistent with previous work (Erzin et al., 2021; Pries et al., 2019, 2020b) and included binary-coded (absent or present) cannabis use, winter-birth, childhood adversity domains (emotional and physical neglect, emotional, physical, and sexual abuse) and bullying. ES-SCZ was calculated by summing log-odds weighted environmental exposures based on our formerly validated estimates (Pries et al., 2019) and a constant of 2 was added to ES-SCZ in accordance with previous reports (Erzin et al., 2021; Pries et al., 2020b) to facilitate interpretation given that an individual might theoretically (although highly unlikely) have a negative value for ES-SCZ if exposed to only physical abuse, which received a negative coefficient in the multivariable prediction model. ES-SCZ: [(cannabis use*1.31) + (winter birth*0.03) + *(emotional abuse*0.78) + (physical abuse*−0.39) + (sexual abuse*0.86) + (emotional neglect*0.44) + (physical neglect*0.25) + (bullying*1.35) + 2]. Details are provided in the online Supplementary file.

### Clinical measures and risk factors

The Positive and Negative Syndrome Scale (PANSS) is a widely used rating scale that consists of 30-items to assess signs and symptoms of schizophrenia across three domains: positive (seven items), negative (seven items) and general psychopathology (16 items) (Kay, Fiszbein, & Opler, 1987). The validated Greek version of PANSS was used (Lykouras, Botsis, & Oulis, 1994). Each item measures the psychopathology severity on a 7-point Likert scale (1 = absent, 2 = minimal, 3 = mild, 4 = moderate, 5 = moderate-severe, 6 = severe, 7 = extreme). The sum scores for each domain thus range between 7 and 49 for the positive and negative symptom domains and between 16 and 112 for the general psychopathology domain. Duration of illness, DUP, and antipsychotic treatment history were evaluated with the Nottingham Onset Schedule (Singh et al., 2005) at baseline using all the available information from family members, health care providers and the medical records. Other environmental and familial risk factors for psychosis included in the present analyses as covariates were the sum score of obstetric and perinatal complications assessed using the Lewis Murray scale (Lewis & Murray, 1987), family history of schizophrenia spectrum disorder assessed using the Family Interview for Genetic Studies (FIGS) (Maxwell, 1992), and ethnicity using native language as a proxy (Greek *v.* others).

### Statistical analyses

Stata software version 16.0 (StataCorp., 2019) was used for all analyses. A *p* value of <0.05 was considered nominally statistically significant. The analyses were limited to baseline and 1-month assessments as the attrition rate at 1-year was 60%. Cross-sectional linear regression analyses were applied to assess the association between ES-SCZ and functioning at baseline and 1-month follow-up. Three models adjusting for (1) demographic variables including age, sex, and education; (2) demographic variables and risk factors including migration status, obstetric complications, ethnicity, and family history of schizophrenia spectrum disorder; and (3) demographic variables and other risk factors and clinical features including PANSS total score (at baseline or 1-month follow-up), previous antipsychotic use and DUP were applied, sequentially. To calculate the relative contribution of the ES-SCZ and each group of covariates (i.e. demographic variables, other risk factors, clinical features) to the *R*^2^ statistic at baseline, the shapely decomposition (Stata shapley2) command was applied to the analyses using the third model. Subsequently, multilevel linear regression analyses were applied to test the longitudinal association between ES-SCZ and functioning as well as clinical features (i.e. PANSS domains). To model the course of functioning and clinical features over time (from baseline to 1-month follow-up) the effects of the ES-SCZ-by-time interaction, ES-SCZ, and time were analyzed (Gueorguieva & Krystal, 2004; Singer & Willett, 2003). Given the hierarchical structure of the data, multilevel mixed-effect models were applied to cluster the multiple assessments per individual. The three above-mentioned models were analyzed sequentially. Of note, when applying model 3, analyses including the PANSS domains as the dependent variable were not adjusted for the PANSS total score.

## Results

Table 1 reports demographic characteristics, clinical features, as well as baseline outcome variables and missing values. Online Supplementary Table S1 reports the frequencies of the exposure components of ES-SCZ, and Table 2 reports assessment of outcome variables at 1-month follow-up.

**Table 1.** Demographic characteristics, clinical features, outcome variables and missing values at baseline

Variables	At baseline	Missing *n* (%)
Age in mean (s.d.)	26.0 (7.6)	1 (0.4%)
Sex		0 (0%)
Male	151 (67.1%)	
Female	74 (32.9%)	
Education years in mean (s.d.)	13.7 (2.5)	3 (1.3%)
Migration		0 (0%)
Yes	30 (13.3%)	
No	195 (86.7%)	
Native language		1 (0.4%)
Greek	216 (96.4%)	
Other	8 (3.6%)	
Antipsychotics		0 (0%)
Yes	77 (34.2%)	
No	148 (65.8%)	
1st-degree family history of psychosis		6 (2.7%)
Yes	33 (15.1%)	
No	186 (84.9%)	
DUP (weeks) in mean (s.d.)	31.3 (63.4)	2 (0.9%)
Obstetric complications in mean (s.d.)	1.3 (3.2)	24 (10.7%)
Any obstetric complications		24 (10.7%)
Present	59 (29.4%)	
Absent	142 (70.6%)	
ES-SCZ in mean (s.d.)	3.8 (1.2)	22 (9.8%)
GAF total in mean (s.d.)	41.3 (15.1)	17 (7.6%)
PSP total in mean (s.d.)	38.0 (15.4)	32 (14.2%)
Socially useful activities	3.6 (1.3)	30 (13.3%)
Personal and social relationships	3.5 (1.2)	30 (13.3%)
Self-care	2.1 (1.4)	30 (13.3%)
Disturbing and aggressive behavior	1.7 (1.5)	30 (13.3%)
PANSS total in mean (s.d.)	97.8 (23.41)	1 (0.4%)
Positive symptoms	28.3 (6.9)	0 (0%)
Negative symptoms	20.2 (8.4)	1 (0.4%)
General symptoms	49.3 (13.6)	0 (0%)

DUP, Duration of untreated psychosis; ES-SCZ, Exposome score for schizophrenia; GAF, The Global Assessment of Functioning scale; PANSS, The Positive and Negative Syndrome Scale; PSP, The Personal and Social Performance scale; s.d., Standard deviation.

**Table 2.** Outcome variables and missing values at 1-month follow-up

	1-month follow-up assessment	Missing *n* (%)
GAF total in mean (s.d.)	60.0 (13.7)	22 (9.8%)
PSP total in mean (s.d.)	57.5 (15.9)	35 (15.6%)
Socially useful activities	2.5 (1.2)	33 (14.7%)
Personal and social relationships	2.4 (1.2)	34 (15.1%)
Self-care	1.2 (1.3)	33 (14.7%)
Disturbing and aggressive behavior	0.5 (0.9)	33 (14.7%)
PANSS total in mean (s.d.)	59.3 (20.0)	2 (0.9%)
Positive symptoms	14.4 (5.9)	2 (0.9%)
Negative symptoms	14.4 (7.0)	2 (0.9%)
General symptoms	30.4 (9.8)	2 (0.9%)

GAF, The Global Assessment of Functioning scale; PANSS, The Positive and Negative Syndrome Scale; PSP, The Personal and Social Performance scale.

### Association between ES-SCZ and functioning at baseline and 1-month follow-up assessments

The investigation of the association between ES-SCZ and GAF at baseline showed that ES-SCZ was associated with the GAF total score in model 1 that was adjusted for age, sex, and education [*B* = −2.29 (95% CI −4.05 to −0.52), *p* value = 0.011]. The results remained significant in model 2 that additionally included other risk factors [*B* = −2.23 (95% CI −4.12 to −0.35), *p* value = 0.021] and in the fully adjusted model 3 that additionally included clinical features [*B* = −2.01 (95% CI −3.88 to −0.13), *p* value = 0.036]. The analyses using the GAF total score at a 1-month follow-up visit as the outcome converged with the results from the baseline data (Table 3), with the exception of the association between ES-SCZ and the GAF total score in model 3 that was not nominally statistically significant. The relative contribution to explained variance (*R*^2^) indicated that model 3 explained 15.2% of the variance in GAF total score at baseline, of which around 38.9% was explained by clinical features, 21.9% by ES-SCZ, 21.4% by other risk factors, and 17.9% by demographic variables (online Supplementary Table S2).

**Table 3.** Association between ES-SCZ and functioning at baseline and 1-month follow-up assessments

	Model 1			Model 2			Model 3		
	Coefficient	95% CI	*p*	Coefficient	95% CI	*p*	Coefficient	95% CI	*p*
Baseline^a^									
GAF total	−2.29	−4.05 to −0.52	**0.011**	−2.23	−4.12 to −0.35	**0.021**	−2.01	−3.88 to −0.13	**0.036**
PSP total	−2.68	−4.61 to −0.75	**0.007**	−2.40	−4.35 to −0.45	**0.016**	−2.31	−4.29 to −0.33	**0.022**
Socially useful activities	0.24	0.08–0.39	**0.003**	0.27	0.11–0.42	**0.001**	0.26	0.11–0.41	**0.001**
Personal and social relationships	0.22	0.07–0.36	**0.003**	0.23	0.08–0.38	**0.003**	0.23	0.08–0.38	**0.003**
Self-care	0.13	−0.04 to 0.29	0.135	0.09	−0.08 to 0.26	0.305	0.07	−0.10 to 0.23	0.436
Disturbing and aggressive behavior	0.08	−0.10 to 0.27	0.373	−0.02	−0.21 to 0.18	0.868	−0.02	−0.21 to 0.17	0.813
1-month follow-up^b^									
GAF total	−2.40	−4.10 to −0.70	**0.006**	−2.50	−4.29 to −0.70	**0.007**	−0.87	−2.43 to 0.70	0.277
PSP total	−2.81	−4.84 to −0.79	**0.007**	−3.00	−5.15 to −0.86	**0.006**	−1.58	−3.56 to 0.39	0.115
Socially useful activities	0.19	0.04–0.35	**0.012**	0.23	0.07–0.39	**0.004**	0.13	−0.02 to 0.27	0.085
Personal and social relationships	0.15	0.00–0.30	**0.048**	0.17	0.01–0.33	**0.040**	0.04	−0.10 to 0.18	0.576
Self-care	0.04	−0.12 to 0.21	0.617	0.02	−0.16 to 0.19	0.853	−0.07	−0.25 to 0.11	0.459
Disturbing and aggressive behavior	−0.01	−0.13 to 0.10	0.819	−0.04	−0.17 to 0.09	0.577	−0.07	−0.20 to 0.07	0.344

CI, Confidence interval; ES-SCZ, Exposome score for schizophrenia; GAF, The Global Assessment of Functioning scale; PSP, The Personal and Social Performance scale.

Model 1 was adjusted for age, sex, and education.

Model 2 was adjusted for age, sex, education, migration, obstetric complications, first language, and family history.

Model 3^a^ was adjusted for age, sex, education, migration, obstetric complications, first language, family history, PANSS total (at baseline), antipsychotic medication, and DUP.

Model 3^b^ was adjusted for age, sex, education, migration, obstetric complications, first language, family history, PANSS total (at 1 month follow up), antipsychotic medication, and DUP.

The investigation of the association between ES-SCZ and PSP at baseline likewise showed that ES-SCZ was associated with the PSP total score in model 1 [*B* = −2.68 (95% CI −4.61 to −0.75), *p* value = 0.007]. The results remained significant in model 2 [*B* = −2.40 (95% CI −4.35 to −0.45), *p* value = 0.016] and in the fully adjusted model 3 [*B* = −2.31 (95% CI −4.29 to −0.33), *p* value = 0.022]. The analyses on functioning at 1-month follow-up confirmed the result (Table 3), except for the association between ES-SCZ and PSP total score in model 3 that was not nominally statistically significant. The relative contribution to explained variance (*R*^2^) indicated that model 3 explained 8.7% of the variance in PSP total score at baseline, of which around 40.9% was explained by ES-SCZ, 29.7% by other risk factors, 22.8% by clinical features, and 6.6% by demographic variables (online Supplementary Table S2). Of note, due to rounding, these numbers diverge from a total sum of 100%.

Subsequent analyses of the PSP subdomains revealed that ES-SCZ was significantly associated with socially useful activities as well as personal and social relationships in all the models, whereas the association with self-care as well as disturbing and aggressive behavior were not statistically significant in either of the models (Table 3). The analyses on the subdomains of PSP at 1-month follow-up confirmed the results, except for the association between ES-SCZ and socially useful activities as well as personal and social relationships in model 3 that were not nominally statistically significant (Table 3). The relative contribution to *R*^2^ (online Supplementary Table S2) indicated that model 3 explained 15.9, 10.5, 17.9, and 12.7% of the variance in PSP socially useful activities, personal and social relationships, self-care, disturbing and aggressive behavior at baseline, respectively. Of this, the relative contribution of ES-SCZ was the largest for the explained variance of socially useful activities (38.2%) and personal and social relationships (53.2%), while the relative contribution of ES-SCZ was the lowest for self-care (4.9%) as well as disturbing and aggressive behavior (0.4%).

### Longitudinal association between ES-SCZ and functioning modeling the trajectories of initial treatment

The longitudinal analyses of the association between ES-SCZ and functioning showed that ES-SCZ was associated with GAF total and PSP total scores in all models (Table 4). Subsequent analyses of the PSP subdomains revealed that ES-SCZ was significantly associated with socially useful activities as well as personal and social relationships in all the models**,** whereas the association with self-care as well as disturbing and aggressive behavior were not statistically significant in either of the models (Table 4).

**Table 4.** Longitudinal association between ES-SCZ and functioning as well as clinical features

	ES-SCZ			Time			Interaction between ES-SCZ and time		
	Coefficient	95% CI	*p*	Coefficient	95% CI	*p*	Coefficient	95% CI	*p*
Model 1									
GAF total	−2.36	−4.05 to −0.67	**0.006**	18.55	12.80–24.30	**<0.001**	0.04	−1.39 to 1.47	0.957
PSP total	−2.69	−4.61 to −0.76	**0.006**	19.82	13.22–26.42	**<0.001**	−0.15	−1.79 to 1.50	0.862
Socially useful activities	0.23	0.08–0.38	**0.003**	−1.02	−1.57 to −0.47	**<0.001**	−0.02	−0.16 to 0.11	0.729
Personal and social relationships	0.21	0.07–0.36	**0.004**	−0.83	−1.35 to −0.32	**0.002**	−0.05	−0.18 to 0.07	0.412
Self-care	0.14	−0.03 to 0.30	0.098	−0.50	−1.05 to 0.05	0.077	−0.10	−0.24 to 0.04	0.149
Disturbing and aggressive behavior	0.09	−0.06 to 0.24	0.260	−0.84	−1.49 to −0.20	**0.010**	−0.10	−0.26 to 0.06	0.203
PANSS	−0.39	−2.91 to 2.12	0.759	−49.13	−59.22 to −39.04	**<0.001**	2.82	0.29–5.35	**0.029**
Positive symptoms	−0.03	−0.78 to 0.71	0.927	−17.09	−20.53 to −13.64	**<0.001**	0.88	0.01–1.74	**0.046**
Negative symptoms	−0.17	−1.06 to 0.71	0.701	−8.50	−10.99 to −6.02	**<0.001**	0.67	0.05–1.30	**0.034**
General symptoms	−0.18	−1.55 to 1.18	0.794	−23.58	−29.23 to −17.93	**<0.001**	1.26	−0.16 to 2.68	0.081
Model 2									
GAF total	−2.28	−4.06 to −0.51	**0.012**	19.34	13.25–25.42	**<0.001**	−0.17	−1.67 to 1.33	0.823
PSP total	−2.43	−4.39 to −0.46	**0.015**	22.12	15.35–28.88	**<0.001**	−0.54	−2.21 to 1.13	0.525
Socially useful activities	0.25	0.10–0.40	**0.001**	−1.13	−1.72 to −0.54	**<0.001**	−0.001	−0.15 to 0.14	0.984
Personal and social relationships	0.21	0.06–0.36	**0.005**	−0.96	−1.51 to −0.41	**0.001**	−0.03	−0.16 to 0.11	0.689
Self-care	0.09	−0.08 to 0.26	0.303	−0.64	−1.23 to −0.05	**0.034**	−0.07	−0.22 to 0.07	0.333
Disturbing and aggressive behavior	0.01	−0.15 to 0.17	0.916	−1.00	−1.68 to −0.32	**0.004**	−0.07	−0.24 to 0.10	0.404
PANSS	0.38	−2.29 to 3.04	0.783	−49.13	−59.72 to −38.55	**<0.001**	2.76	0.13–5.39	**0.040**
Positive symptoms	0.08	−0.69 to 0.86	0.836	−17.00	−20.66 to −13.34	**<0.001**	0.85	−0.06 to 1.76	0.067
Negative symptoms	0.06	−0.88 to 1.00	0.898	−8.76	−11.45 to −6.06	**<0.001**	0.70	0.03–1.37	**0.040**
General symptoms	0.23	−1.20 to 1.67	0.748	−23.39	−29.20 to −17.59	**<0.001**	1.20	−0.24 to 2.65	0.102
Model 3									
GAF total^a^	−1.93	−3.60 to −0.26	**0.023**	8.93	2.62–15.23	**0.006**	0.42	−0.97 to 1.80	0.558
PSP total^a^	−2.30	−4.19 to −0.40	**0.018**	13.88	6.57–21.18	**<0.001**	−0.09	−1.72 to 1.53	0.910
Socially useful activities ^a^	0.24	0.10–0.39	**0.001**	−0.47	−1.11 to 0.16	0.143	−0.04	−0.18 to 0.10	0.592
Personal and social relationships ^a^	0.21	0.06–0.35	**0.004**	−0.38	−0.99 to 0.23	0.219	−0.06	−0.20 to 0.07	0.383
Self-care ^a^	0.06	−0.10 to 0.23	0.440	−0.17	−0.83 to 0.50	0.621	−0.10	−0.25 to 0.05	0.189
Disturbing and aggressive behavior^a^	−0.01	−0.16 to 0.15	0.941	−0.28	−0.98 to 0.42	0.427	−0.11	−0.27 to 0.04	0.161
PANSS ^b^	0.34	−2.32 to 3.01	0.802	−49.01	−59.60 to −38.41	**<0.001**	2.75	0.12–5.39	**0.041**
Positive symptoms^b^	0.11	−0.66 to 0.87	0.783	−16.96	−20.63 to −13.29	**<0.001**	0.85	−0.06 to 1.76	0.068
Negative symptoms^b^	0.01	−0.93 to 0.95	0.978	−8.71	−11.41 to −6.02	**<0.001**	0.70	0.03–1.37	**0.041**
General symptoms^b^	0.22	−1.21 to 1.66	0.761	−23.35	−29.16 to −17.53	**<0.001**	1.20	−0.25 to 2.65	0.104

CI, Confidence interval; ES-SCZ, Exposome score for schizophrenia; GAF, The Global Assessment of Functioning scale; PANSS, The Positive and Negative Syndrome Scale; PSP, The Personal and Social Performance scale.

Model 1 was adjusted for age, sex, and education.

Model 2 was adjusted for age, sex, education, migration, obstetric complications, first language, and family history.

a Model 3 was adjusted for age, sex, education, migration, obstetric complications, first language, family history, PANSS total, antipsychotic medication, and DUP.

b Model 3 was adjusted for age, sex, education, migration, obstetric complications, first language, family history, antipsychotic medication, and DUP.

The analyses of change of GAF total and PSP total scores over time indicated that functioning increased from baseline assessment to the 1-month follow-up assessment (Table 4). The analyses of the PSP subdomains (i.e. socially useful activities, personal and social relationships, self-care, disturbing and aggressive behavior) converged with the results from the total scores and showed that the severity of the subdomains decreased from baseline to the 1-month follow-up assessment, with the exception of self-care in model 1, which was trend significant; and the subdomain analyses in model 3, which were not nominally statistically significant (Table 4).

**Fig. 1.**
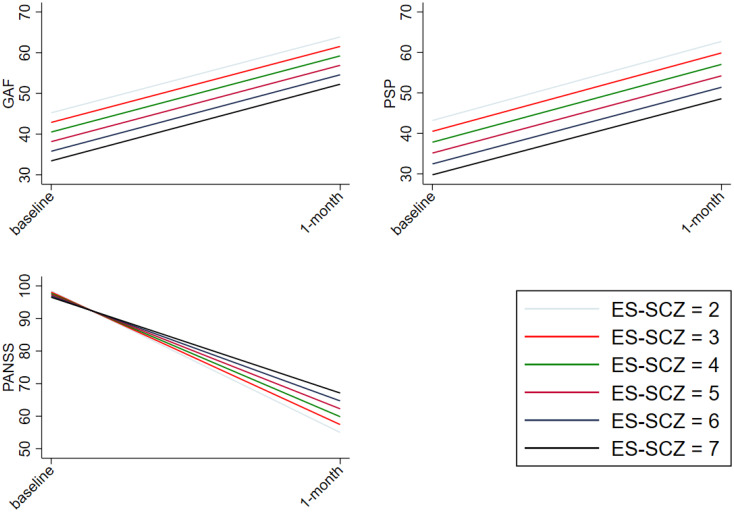
Longitudinal association between ES-SCZ and functioning as well as clinical features. shows the marginal plots of the interaction between the exposome score for schizophrenia (ES-SCZ) and functioning (GAF, The Global Assessment of Functioning scale; PSP, The Personal and Social Performance scale) as well as clinical features (PANSS, The Positive and Negative Syndrome Scale) adjusted for age, sex and education.

No significant ES-SCZ-by-time interaction was found on functioning (GAF and PSP) in either of the models, meaning that changes in functioning were not dependent on ES-SCZ (see Fig. 1 for marginal plots of the analyses in model 1). The analyses on PSP subdomains (i.e. socially useful activities, personal and social relationships, self-care, disturbing and aggressive behavior) converged with the results from the total scores (Table 4).

### Longitudinal association between ES-SCZ and clinical features modeling the trajectories of initial treatment

The association between ES-SCZ and PANSS scores (i.e. total, negative, positive, and general symptoms) were not statistically significant in either of the models (Table 4). However, the analyses of change of PANSS subdomains over time indicated that clinical features decreased from baseline to the 1-month follow-up assessment in all the models (Table 4).

The analyses of the PANSS total score over time indicated an ES-SCZ-by-time interaction in model 1 [*B* = 2.82 (95% CI 0.29–5.35), *p* value = 0.029], meaning that the decrease of the total score over time were dependent on ES-SCZ and individuals with high ES-SCZ showed less improvement (see Fig. 1 for the marginal plot of the analyses in model 1). The results remained significant in model 2 [*B* = 2.76 (95% CI 0.13–5.39), *p* value = 0.040] and in the fully adjusted model 3 [*B* = 2.75 (95% CI 0.12–5.39), *p* value = 0.041] (Table 4). Furthermore, subsequent analyses of the PANSS subdomains (i.e. negative, positive, general symptoms) revealed that there were ES-SCZ-by-time interactions on negative symptoms in model 1 [*B* = 0.67 (95% CI 0.05–1.30), *p* value = 0.034], in model 2 [*B* = 0.70 (95% CI 0.03–1.37), *p* value = 0.040], and in model 3 [*B* = 0.70 (95% CI 0.03–1.37), *p* value = 0.041]. Furthermore, there was an ES-SCZ-by-time interaction on positive symptoms in model 1 [*B* = 0.88 (95% CI 0.01–1.74), *p* value = 0.046]. The results became trend significant in model 2 (*B* = 0.85 (95% CI −0.06 to 1.76), *p* value = 0.067] and in the fully adjusted model 3 [*B* = 0.85 (95% CI −0.06 to 1.76), *p* value = 0.068] (Table 4). The interaction between ES-SCZ and time on general symptoms was trend significant in model 1 [*B* = 1.26 (95% CI −0.16 to 2.68), *p* value = 0.081]. No other statistically significant interactions were found (Table 4).

## Discussion

This study investigated the cross-sectional and temporal associations of ES-SCZ with global functioning, personal and social functionality and symptom severity in an FEP cohort of mainly antipsychotic-naïve patients. We demonstrated that ES-SCZ was associated with overall functioning, specifically, the domains of socially useful activities and personal/social relationships but not the domains of self-care and disturbing behavior at baseline and 1-month assessments, respectively. These findings remained significant even when accounting for several associated alternative explanatory variables including other environmental exposures that were previously associated with psychosis spectrum disorder, clinical characteristics such as DUP and symptom severity, as well as the family history of psychosis, demonstrating that the association between ES-SCZ and functioning is over and above other risk factors and cannot be explained by symptom severity solely. Our longitudinal analysis investigating the change in functioning and symptom severity from baseline to 1-month assessment revealed evidence for an association of ES-SCZ with improvement in psychotic symptoms, in particular negative symptoms. These findings suggest that ES-SCZ appears to be a stable trait indicator of poor functioning across time points and is related to the trajectory of symptomatic improvement but not functioning.

To the best of our knowledge, this is the first study examining the relationship of ES-SCZ with symptomatic and functional outcomes in individuals with FEP. Our findings replicate the results of a recent study showing a strong and consistent association between ES-SCZ and functioning domains (GAF symptom and disability dimensions) in individuals diagnosed with schizophrenia, as well as in their siblings, and healthy controls (Erzin et al., 2021). Our current results also echo findings of previous research showing that ES-SCZ was not only related to clinical schizophrenia diagnosis and continuous schizotypy traits (Pries et al., 2020b) but also temporally associated with poor general mental health outcomes (Pries et al., 2020a, 2021b), providing further support to the utility of ES-SCZ for determining the degree of mental well-being. Furthermore, we demonstrated that even when accounting for family history of psychotic disorders as a proxy of genetic liability, as well as several other known environmental risk factors of psychosis that were not included in ES-SCZ (i.e. obstetric complications, ethnic minority status, and migration), the relation between ES-SCZ and functioning remained nominally statistically significant, with no reduction in the magnitude of the association.

These findings are in line with recent research and confirm that the relation between ES-SCZ and functioning cannot be reduced to either genetic liability for schizophrenia or other environmental vulnerability that is not captured by ES-SCZ (Erzin et al., 2021). However, approaches that capture genetic vulnerability through a single metric of molecular genetic risk such as the polygenic risk score for schizophrenia (PRS-SCZ), may moderate the effects of ES-SCZ on functioning in FEP. PRS-SCZ was recently associated with illness course and treatment effects in FEP (Zhang et al., 2019). Furthermore, studies found positive additive interaction effects between PRS-SCZ and cumulative environmental vulnerability for schizophrenia on the risk of schizophrenia, FEP, as well as schizotypy in siblings of patients and healthy controls (Guloksuz et al., 2019; Mas et al., 2020; Pries et al., 2020b). Following this research line, future studies are required to further investigate whether PRS-SCZ moderates the association between ES-SCZ and functioning across the psychosis spectrum and evaluate the potential combined value of ES-SCZ and PRS-SCZ.

Our findings derived from the final adjusted model including DUP, symptom severity, and previous exposure to antipsychotics showed that ES-SCZ explained functioning over and above clinical representation exclusively. This was also supported by the evaluation of the relative contribution to the explained variance (R^2^) of functioning. ES-SCZ was the largest contributor for PSP total score, PSP socially useful activities, as well as for PSP personal and social relationships. Furthermore, it was the second-largest contributor to GAF total scores following the clinical features. Our findings highlight the possible utility of assessing exposomic vulnerability in addition to clinical features to estimate potential illness trajectories within clinical settings. A recent study using the current dataset, demonstrated that a clinical prediction model including clinical features and other correlates of psychosis significantly predicted remission at a 1-month follow-up (Soldatos et al., 2021). It is possible that the integration of ES-SCZ into these and similar models may further improve prediction performance in future studies.

Our mixed model regression analyses showed that there was a statistically significant improvement in total functioning scores (both GAF and PSP) from baseline to 1-month assessment, with an increase in functioning across all PSP domains except the self-care category. Similar to the baseline results, ES-SCZ was associated with functioning scores at 1-month in all models but not with changes in functioning over time. In contrast, ES-SCZ was not associated with symptom severity at baseline but improvement in symptom severity from baseline to 1-month assessment, particularly the negative symptom domain. In the light of present findings and combined with previous evidence from the Dutch general population cohort study (Guloksuz et al., 2020; Pries et al., 2021b) and EUGEI (Erzin et al., 2021), it is plausible to argue that rather than a predictor of functional improvement, ES-SCZ represents a severity indicator that captures poor functioning in psychosis spectrum disorder. In this respect, ES-SCZ can be beneficial for clinical characterization (Maj et al., 2021) and incorporated into transdiagnostic staging models (Shah et al., 2020).

Our findings show that the total environmental predisposition to schizophrenia (ES-SCZ) is not only associated with psychosis but also with the short-term illness course. However, long-term follow-up analyses are needed to investigate the stability of the current results given that FEP trajectories diverge over time. In accordance with our findings, previous research investigating exposures individually, such as childhood adversities, has consistently shown that childhood adversities including bullying are associated with more severe illness courses (Alameda et al., 2016; Schalinski, Fischer, & Rockstroh, 2015) lower rates of remission, worse outcomes (Thomas, Höfler, Schäfer, & Trautmann, 2019), and poor functioning in early psychosis (Alameda et al., 2015; Pruessner et al., 2021; Yung et al., 2015). Similarly, premorbid cannabis use before the onset of psychosis has been associated with symptom severity and poorer functioning, and this association was independent of present substance use and premorbid functioning (Ringen et al., 2016). Furthermore, an aggregate environmental risk score was associated with a transition to clinical psychosis in youth at familial high risk for psychosis (Padmanabhan et al., 2017). Taken together, these findings reinforce the notion that environmental exposures account for symptom severity and poorer functioning in psychosis.

### Methodological considerations

There were several strengths and limitations of our study. First, ES-SCZ was constructed based on the information collected using the same assessment tools and the definitions that were identical to those applied in the study that estimated the risk of each exposure in ES-SCZ, with the exception of hearing impairment (Erzin et al., 2021; Pries et al., 2019, 2020b). However, it is reasonable to presume that had the hearing impairment been measured, our findings would be even more pronounced. Second, ES-SCZ may be further enriched by the inclusion of other exposures that have been previously associated with psychosis such as the use of other substances (e.g. tobacco), ethnic minority, migration, or pre-, perinatal period adversities. Accumulating evidence suggests that tobacco use might be associated with schizophrenia (Gurillo, Jauhar, Murray, & MacCabe, 2015; Hunter, Murray, Asher, & Leonardi-Bee, 2020), with findings from Mendelian Randomization studies suggesting a possible causal link (Treur, Munafò, Logtenberg, Wiers, & Verweij, 2021). However, a major challenge of integrating tobacco use into a collective environmental liability index, such as the ES-SCZ, would be the very high correlation between cannabis use and tobacco consumption. The current findings suggest that the degree of the association between ES-SCZ and functioning is not reduced when controlling for other environmental factors such as obstetric complications, ethnic minority, and migration, the inclusion of these additional exposures may nevertheless increase the predictive power of ES-SCZ. However, it should be noted that obstetric complications are rarely available in ongoing cohort samples and extremely challenging to assess reliably in retrospect without detailed birth records. Furthermore, ethnic minority and migration were not included in ES-SCZ as this would diminish the application of ES-SCZ in different populations worldwide with lower diversity (Burkhard, Cicek, Barzilay, Radhakrishnan, & Guloksuz, 2021; Pries et al., 2021b), as well as decrease the utility of ES-SCZ when combined with PRS-SCZ in heterogenous samples given the low performance of PRS-SCZ in diverse samples (Bigdeli et al., 2020). Third, we analyzed both the identical functioning assessment (GAF) to enable a true replication of the previous study (Erzin et al., 2021) and a more detailed functioning assessment (PSP) to confirm and extend our findings showing an association between ES-SCZ and functioning in a longitudinal FEP cohort. However, our analysis was limited to a relatively short follow-up duration (baseline and 1-month assessments) as the attrition rate at 1-year was 60%. Future studies should conduct long-term follow-up replications to examine the stability of our findings across time.

Fourth, deep clinical phenotyping and rich environmental assessment allowed for inclusion of several associated alternative explanatory variables to test the robustness of the association between ES-SCZ and functioning independent of clinical and other environmental features, such as pre- and perinatal risk factors (Davies et al., 2020; Paquin, Lapierre, Veru, & King, 2021). However, there might still be unmeasured confounding considering the extensive list of factors associated with functioning. Finally, the confounding of antipsychotic use was negligible, as the majority of the study participants (66%) was medication-naïve at baseline, with the remaining subjected to only minimal antipsychotic exposure. Although this was a relatively large FEP cohort, larger samples might be required to analyze the mediators and moderators of the association between environmental predisposition to schizophrenia and functioning.

## Conclusions

Our findings provide further support to the notion that environmental risk loading for schizophrenia (ES-SCZ) may aid in estimating the functioning over the course of a psychotic disorder (Erzin & Guloksuz, 2021; Pries, Erzin, Rutten, van Os, & Guloksuz, 2021a). Furthermore, our findings suggest that the influence of ES-SCZ on functioning outcomes cannot be reduced to the impact of other known risk factors for schizophrenia or clinical characteristics. In this regard, the assessment of environmental risk factors for schizophrenia should be an integral part of the routine clinical evaluation in individuals with FEP.

## Supplementary material

For supplementary material accompanying this paper visit https://doi.org/10.1017/S0033291721004542. click here to view supplementary material
