# Supplementary material for: Association between exposome score for schizophrenia and functioning in first-episode psychosis: results from the Athens first-episode psychosis research study
Source: Psychol Med. 2021 Nov 18;53(6):2609–18. doi: 10.1017/S0033291721004542 (PMC10123830; doi:10.1017/S0033291721004542)
Supplement: Supplementary file 1 [file S0033291721004542sup001.docx]

**Supplementary Methods. Calculations and Measures**

**The estimation of exposome score for schizophrenia**

With exception of hearing impairment, which was not collected in the Athens FEP Research Study, the assessments and definitions of environmental exposures were consistent with previous work (Erzin et al., 2021; Pries et al., 2020; Pries et al., 2019) and included binary-coded (absent or present) cannabis use, winter-birth, childhood adversity domains (emotional and physical neglect, emotional, physical, and sexual abuse) and bullying. ES-SCZ was calculated by summing log-odds weighted environmental exposures based on our formerly validated estimates (Pries et al., 2019) and a constant of 2 was added to ES-SCZ in accordance with previous reports (Erzin et al., 2021; Pries et al., 2020) to facilitate interpretation given that an individual might theoretically (although highly unlikely) have a negative value for ES-SCZ if exposed to only physical abuse, which received a negative coefficient in the multivariable prediction model. ES-SCZ: ((cannabis use*1.31) + (winter birth*0.03) +*(emotional abuse*0.78) + (physical abuse*-0.39) + (sexual abuse*0.86) + (emotional neglect*0.44) + (physical neglect*0.25) + (bullying*1.35) + 2).

The CTQ was used for assessing childhood adversity (Bernstein et al., 2003). This form includes 28 items, and these items are rated on a 5-point Likert scale which measure five domains of maltreatment. These domains are emotional, physical, and sexual abuse, emotional and physical neglect. To dichotomize each childhood adversity domain as 0=“absent” and 1=“present” in accordance with previous work  (Erzin et al., 2021; Guloksuz et al., 2019; Kraan et al., 2018; Pries et al., 2020). The cut-off scores are ≥ 9 for emotional abuse; ≥6 for sexual abuse; ≥8 for physical abuse; ≥8 for physical neglect; ≥10 for emotional neglect.

The Cannabis Experiences Questionnaire was used for measuring cannabis use. The Cannabis Experiences Questionnaire (0=“none”; 1=“only once or twice”; 2=“a few times a year”; 3=“a few times a month”; 4=“once or more a week”; 5=“everyday”) is Likert type scale. Based on previous work (Guloksuz et al., 2019; Pries et al., 2018; Pries et al., 2020; Radhakrishnan et al., 2019; Van Winkel, 2011) a binary cannabis use variable occurred. If cannabis use is one or more per week during the lifetime period, cannabis use is accepted as ‘Yes’.

In line with previous studies investigating the relationship between the birth season and schizophrenia spectrum disorder in Northern Hemisphere (Davies, Welham, Chant, Torrey, & McGrath, 2003), the high-risk birth period was the winter solstice (December-March). By using the cut-off point ≥1, childhood bullying was dichotomized as 0= “absent” and ≥1=“present”, conforming to previous studies (Guloksuz et al., 2019; Pries et al., 2019; Pries et al., 2020).

**Antipsychotic medication**

At baseline, 34% of the individuals with first episode psychosis were on antipsychotics, whereas this rate was 95.5% at 1-month follow-up. The mean chlorpromazine equivalent dose of the antipsychotic treatment was 402.4 at 1-month follow-up (SD = 225.2).

**Supplementary table** S1 Environmental exposures and missing values at baseline

| Variables | At baseline n (%) | Missing n (%) |
| --- | --- | --- |
| Cannabis use |  | 7 (3.1%) |
| Yes | 71 (32.6%) |  |
| No | 147 (67.4%) |  |
| Winter-birth |  | 6 (2.7%) |
| Yes | 79 (36.1%) |  |
| No | 140 (63.9%) |  |
| Physical abuse |  | 5 (2.2%) |
| Yes | 34 (15.5%) |  |
| No | 186 (84.6%) |  |
| Emotional abuse |  | 5 (2.2%) |
| Yes | 87 (39.6%) |  |
| No | 133 (60.5%) |  |
| Physical neglect |  | 4 (1.8%) |
| Yes | 62 (28.1%) |  |
| No | 159 (72.0%) |  |
| Sexual abuse |  | 7 (3.1%) |
| Yes | 34 (15.6%) |  |
| No | 184 (84.4%) |  |
| Emotional neglect |  | 6 (2.7%) |
| Yes | 113 (51.6%) |  |
| No | 106 (48.4%) |  |
| Bullying |  | 3 (1.3%) |
| Yes | 114 (51.4%) |  |
| No | 108 (48.7%) |  |

|  | GAF total (%) | PSP total  (%) | PSP Socially useful activities (%) | PSP Personal and social relationships (%) | PSP Self-care (%) | PSP Disturbing and aggressive behavior (%) |
| --- | --- | --- | --- | --- | --- | --- |
| ES-SCZ | 3.3 | 3.6 | 6.1 | 5.6 | 0.9 | 0.0 |
| Demographic variables | 2.7 | 0.6 | 0.8 | 0.7 | 3.4 | 1.8 |
| Other risk factors | 3.2 | 2.6 | 6.3 | 2.3 | 3.2 | 4.9 |
| Clinical features | 5.9 | 2.0 | 2.7 | 1.9 | 10.5 | 5.9 |
| Total^a^ | 15.2 | 8.7 | 15.9 | 10.5 | 17.9 | 12.7 |

**Supplementary table S2** Contributions of ES-SCZ and covariate groups to functioning

^a^ Total contribution to R2 by ES-SCZ, demographic variables (i.e., age, sex, and education), other risk factors (i.e. migration status, obstetric complications, ethnicity, and family history of schizophrenia spectrum disorder), and clinical features (i.e. Positive and Negative Syndrome Scale [PANSS] total score at baseline, antipsychotic medication, and duration of untreated psychosis [DUP]); ES-SCZ: Exposome score for schizophrenia, GAF: The Global Assessment of Functioning, PSP: Personal and Social Performance Scale; R2: Explained variance

**References**

Bernstein, D. P., Stein, J. A., Newcomb, M. D., Walker, E., Pogge, D., Ahluvalia, T., . . . Zule, W. (2003). Development and validation of a brief screening version of the Childhood Trauma Questionnaire. *Child Abuse & Neglect, 27*(2), 169-190. doi:10.1016/s0145-2134(02)00541-0

Davies, G., Welham, J., Chant, D., Torrey, E. F., & McGrath, J. (2003). A Systematic Review and Meta-analysis of Northern Hemisphere Season of Birth Studies in Schizophrenia. *Schizophrenia Bulletin, 29*(3), 587-593. doi:10.1093/oxfordjournals.schbul.a007030

Erzin, G., Pries, L.-K., Van Os, J., Fusar-Poli, L., Delespaul, P., Kenis, G., . . . Guloksuz, S. (2021). Examining the association between exposome score for schizophrenia and functioning in schizophrenia, siblings, and healthy controls: Results from the EUGEI study. *European Psychiatry, 64*(1), 1-33. doi:10.1192/j.eurpsy.2021.19

Guloksuz, S., Pries, L. K., Delespaul, P., Kenis, G., Luykx, J. J., Lin, B. D., . . . Van Os, J. (2019). Examining the independent and joint effects of molecular genetic liability and environmental exposures in schizophrenia: results from the EUGEI study. *World Psychiatry, 18*(2), 173-182. doi:10.1002/wps.20629

Kraan, T. C., Velthorst, E., Themmen, M., Valmaggia, L., Kempton, M. J., McGuire, P., . . . Van Der Gaag, M. (2018). Child Maltreatment and Clinical Outcome in Individuals at Ultra-High Risk for Psychosis in the EU-GEI High Risk Study. *Schizophrenia Bulletin, 44*(3), 584-592. doi:10.1093/schbul/sbw162

Pries, L.-K., Guloksuz, S., Ten Have, M., De Graaf, R., Van Dorsselaer, S., Gunther, N., . . . Bak, M. (2018). Evidence that environmental and familial risks for psychosis additively impact a multidimensional subthreshold psychosis syndrome. *Schizophrenia bulletin, 44*(4), 710-719.

Pries, L.-K., Lage-Castellanos, A., Delespaul, P., Kenis, G., Luykx, J. J., Lin, B. D., . . . Guloksuz, S. (2019). Estimating Exposome Score for Schizophrenia Using Predictive Modeling Approach in Two Independent Samples: The Results From the EUGEI Study. *Schizophrenia Bulletin, 45*(5), 960-965. doi:10.1093/schbul/sbz054

Pries, L. K., Dal Ferro, G. A., Van Os, J., Delespaul, P., Kenis, G., Lin, B. D., . . . Guloksuz, S. (2020). Examining the independent and joint effects of genomic and exposomic liabilities for schizophrenia across the psychosis spectrum. *Epidemiology and Psychiatric Sciences, 29*. doi:10.1017/s2045796020000943

Radhakrishnan, R., Guloksuz, S., Ten Have, M., De Graaf, R., Van Dorsselaer, S., Gunther, N., . . . Van Os, J. (2019). Interaction between environmental and familial affective risk impacts psychosis admixture in states of affective dysregulation. *Psychological Medicine, 49*(11), 1879-1889. doi:10.1017/s0033291718002635

Van Winkel, R., Risk, G., & Outcome of Psychosis (GROUP) Investigators (2011). Family-based analysis of genetic variation underlying psychosis-inducing effects of cannabis: sibling analysis and proband follow-up. *Archives of general psychiatry*, *68*(2), 148-157.
